# Surgery

**Published:** 1905-06

**Authors:** Chas. A. Morton


### SURGERY.

**Surgical Shock.**—Why does a person die after a severe operation unattended by serious hemorrhage? Mr. Lockhart Mummery, in his recent Hunterian Lectures[2] at the College of Surgeons, has endeavoured to answer this question, and to indicate the treatment likely to prevent the tendency to death from this condition. He says he is afraid he has but swept away some of the dust from the doorstep of a great subject, but he hopes he may encourage others to look further into its mysteries; and he admits that the test of time and experience is necessary before the methods of treatment proposed can be accepted as of proved value. Nevertheless, he has gathered together and presented to us in these lectures information of much value.

How many theories have been advanced to explain this condition of surgical shock, and how little we know of the cause of the tendency to death in many other conditions. Very often the heart is already failing, when the operation comes "as the last straw" and it quickly fails; but it is often as difficult to say why it was failing from the condition of disease present before the operation as to explain the acceleration of the fatal termination by the operation itself. Take as an instance a case of acute intestinal obstruction. What is it that is impairing

[1] *Am. Med.*, 1905, ix. 519. [2] *Lancet*, 1905, i. 696, 776, 846.

the cardiac power? Is it a toxine absorbed from the retained intestinal contents? If so, why does a patient die so much more quickly with a strangulated coil than with an absolute obstruction due to growth? Possibly the strangulation may, through the sympathetic nerves, cause a condition of dilatation of the veins in the splanchnic area, and so produce a condition analogous to what many believe to be present in surgical shock.

Mr. Lockhart Mummery's conclusions as to the pathology and treatment of shock are mainly based on the experimental work of the American surgeon Crile, whose book on the subject is of course well known, and was reviewed in this *Journal* in 1899.[1] Shock must clearly act on the heart through the nervous system, and the only direct way it could paralyse the heart would be through the vagi; and there is reason to believe that in some operations in the area supplied by the superior laryngeal nerve there is a direct inhibitory action on the heart, and in the ordinary condition of shock, such as would be produced by severe crushing of a limb, there might be a reflex vagus inhibition; but that shock is not due to nervous influence directly on the heart, Crile has shown by experimentally producing shock after the cardiac branches of the stellate ganglion had been divided, or the vagi divided. The heart must fail then through some effect on the circulation, and that this is a fall in blood pressure from exhaustion of the vaso-motor centre Crile considers his experiments have established, and in this conclusion Lockhart Mummery and others agree. This of course is no new theory, as Crile himself allows.

Crile has shown that shock from injuries of, or operations on, the extremities can be entirely prevented by blocking the main nerves by injecting cocaine into them, and this is a method which should certainly be adopted in amputations high up in either the upper or lower limbs. The first effect of injury to any part is a rise in blood pressure, and then as the injury is repeated, instead of a rise, the blood pressure begins to fall, and when the injury is very severe or prolonged, it falls to so great an extent that sufficient blood does not reach the heart, and the circulation fails. It is believed that a large portion of the blood lies in the dilated abdominal veins. Experiments have shown that merely exposing the intestines, and still more manipulation of them, will cause a fall of blood pressure, and that this is due to the blood collecting in the dilated veins of the splanchnic area is proved by the fact that if the arteries supplying this area are clamped, exposure and manipulation of the intestines have no such effect. Lockhart Mummery's own observations on the blood pressure during abdominal operations, made by means of a recording instrument attached to the surface of the limb over a large artery, show[2] that at first the pressure rises, and only when the manipulation is prolonged does a fall occur. Evisceration seems to produce a very dangerous degree of fall

[1] Page 353. [2] *Loc. cit.*, p. 777.

in the blood pressure. The application of heat to the peritoneum may cause either a rise or a fall; if the latter, it usually occurred in the later stages of the operation. He gives many instances of a fall in blood pressure during operations of various kinds, but he does not indicate whether symptoms of shock developed in connection with the fall in pressure.

There is a great difference in the effect on the blood pressure of ether or chloroform as an anæsthetic. The former causes a rise, the latter a fall. Hence in operations likely to lead to shock ether is clearly indicated. Indeed, it seems possible that the occasional serious effects of chloroform may be due very largely to its causing a dangerous fall in blood pressure. Anæsthesia produced by spinal cocainisation causes a fall in blood pressure from paralysis of the vaso-motor nerves, by which the splanchnic area is controlled, and would thus increase the tendency to shock.

It has seemed to me very difficult to understand why a person with a crushed cervical cord does not die of shock, if it is essentially a breakdown of the vaso-motor mechanism, for the whole body must be cut off from the vaso-motor centre. I have seen patients, soon after the injury, presenting no signs of shock at all. Landois, in his *Physiology*, however, states that if the cord is severed high up general vaso-motor paralysis results, but in "a few days" secondary vaso-motor centres in the cord take on the work.

In the treatment of shock we have placed great reliance on the administration of alcohol and strychnine. It is somewhat startling to learn that they are both broken reeds, or even worse, harmful agents. We have been inclined to give larger and larger doses of strychnine, only dreading convulsive effects; but we are told on Crile's experimental evidence that by so doing a condition of profound shock may actually be produced, without causing convulsions, from exhaustion of the vaso-motor centre from excessive stimulation. Strychnine does raise the blood pressure, but a worse fall follows the rise. Alcohol injected into the veins neither increased the blood pressure (it more often caused some fall) nor stimulated the heart to more forcible action; and, in condition of severe shock, increased it by further lowering the blood pressure.

If then strychnine and alcohol must be regarded as useless in the treatment of shock, on what can we rely in dealing with the condition? The intravenous injection of adrenalin seems one of the most useful methods we can employ, but it must be intravenous, given as we should saline infusion, but never stronger than 1 in 20,000, and only at the rate of about three to five cubic centimetres a minute. It should be continued until on stopping it the blood pressure is found to remain at a safe level, for the blood pressure may soon fall when the infusion is discontinued in severe cases. It would be interesting to know for how long life could be prolonged in this way, if the vasomotor centre did not recover its functions. Perhaps the most certain way of increasing the blood pressure in conditions of shock is to increase the pressure on the surface of the body, or at any rate with the exception of the head and chest. Crile had a pneumatic suit made, which enclosed the body below the chest, and was inflated by a foot pump. By the use of this suit he was able so to raise the blood pressure in shock that he saved at any rate one life that would otherwise have been lost. But, although such pneumatic suits may not be available, there are other methods which, acting to some extent in the same way, may prove of value. The head and chest should be placed on a lower level than the lower limbs and the abdomen by raising the foot of the bed, and the lower limbs may be compressed by bandages. Pressure on the abdomen, too, is a very valuable method of raising the blood pressure by lessening the amount of blood in the abdominal vessels, and such pressure may well be maintained by the use of a tight abdominal binder over a mass of cotton wool. Whether saline infusion into the veins does good in shock, as it undoubtedly does in hemorrhage, has been a much disputed question. Crile's experiments and Lockhart Mummery's clinical observations on blood pressure in shock, show that it can very considerably raise the blood pressure, but that if the condition of shock is profound the pressure very soon falls again. It is therefore of only very limited value, as it cannot be used in indefinite quantity, for this would only result in general œdema of the tissues. Still it is of temporary value, and it is easy to leave a pint or two of saline solution in the peritoneal cavity at the end of an abdominal operation, and this will be readily absorbed, and tend to raise the blood pressure. If there is a general septic infection of the peritoneal cavity, it will do good also by stimulating leucocytosis and diluting the septic toxines.

CHARLES A. MORTON.

